# The long-term effects of cash transfer programmes on young adults’ mental health: a quasi-experimental study of Colombia, Mexico, and South Africa

**DOI:** 10.1093/heapol/czae102

**Published:** 2024-11-01

**Authors:** Annie Zimmerman, Mauricio Avendano, Crick Lund, Ricardo Araya, Yadira Diaz, Juliana Sanchez-Ariza, Philipp Hessel, Emily Garman, Sara Evans-Lacko

**Affiliations:** Health Service & Population Research Department, Centre for Global Mental Health, King’s College London, Institute of Psychiatry, Psychology and Neuroscience, de Crespigny Park, London SE5 8AF, United Kingdom; King’s College London, Global Health & Social Medicine, 40 Aldwych, London WC2R 2LS, United Kingdom; Department of Epidemiology and Health Systems, Center for Primary Care and Public Health (Unisanté), University of Lausanne, 10 route de la Corniche, Lausanne 1010, Switzerland; Health Service & Population Research Department, Centre for Global Mental Health, King’s College London, Institute of Psychiatry, Psychology and Neuroscience, de Crespigny Park, London SE5 8AF, United Kingdom; Department of Psychiatry and Mental Health, Alan J Flisher Centre for Public Mental Health, University of Cape Town, 46 Sawkins Rd, Rondebosch, Cape Town 7700, South Africa; Health Service & Population Research Department, Centre for Global Mental Health, King’s College London, Institute of Psychiatry, Psychology and Neuroscience, de Crespigny Park, London SE5 8AF, United Kingdom; Escuela de Gobierno Alberto Lleras Camargo, Universidad de Los Andes, Carrera 1° N° 19-27, Bloque AU, piso 2, Bogotá 111711, Colombia; Escuela de Gobierno Alberto Lleras Camargo, Universidad de Los Andes, Carrera 1° N° 19-27, Bloque AU, piso 2, Bogotá 111711, Colombia; Escuela de Gobierno Alberto Lleras Camargo, Universidad de Los Andes, Carrera 1° N° 19-27, Bloque AU, piso 2, Bogotá 111711, Colombia; Department of Epidemiology and Public Health, Swiss Tropical and Public Health Institute, Socinstrasse 5, Basel 4051, Switzerland; Department of Psychiatry and Mental Health, Alan J Flisher Centre for Public Mental Health, University of Cape Town, 46 Sawkins Rd, Rondebosch, Cape Town 7700, South Africa; Department of Health Policy, Care Policy and Evaluation Centre, London School of Economics and Political Science, Houghton Street, London WC2A 2AE, United Kingdom

**Keywords:** poverty, cash transfer programme, global mental health, youth mental health, quasi-experimental, social policy, low- and middle-income countries

## Abstract

Poverty is associated with poorer mental health in early adulthood. Cash transfers (CTs) have been shown to improve child health and education outcomes, but it is unclear whether these effects may translate into better mental health outcomes as children reach young adulthood. Using a quasi-experimental approach that exploits variation across countries in the timing of national CT programme introduction, we examine whether longer exposure to CTs during childhood (0–17 years) reduces depressive symptoms in early adulthood (18–30 years). Based on harmonized data from Colombia, Mexico, and South Africa (*N* = 14 431), we applied logistic regression models with country and birth-cohort fixed effects to estimate the impact of cumulative years of CT exposure on mental health, educational attainment, and employment outcomes. Our findings indicate that each additional year of CT exposure during childhood is associated with a 4% reduction in the odds of serious depressive symptoms in early adulthood [odds ratio (OR) = 0.96, 95% confidence intervals (CIs): 0.93, 0.98]. We find no consistent effect of years of exposure on completion of secondary school (OR = 1.01, 95% CIs: 0.99, 1.03) and a negative effect on the probability of employment in early adulthood (OR = 0.90, 95% CIs: 0.88, 0.91). These results suggest that longer exposure to CTs may contribute to modest but meaningful reductions in population-level depressive symptoms during early adulthood.

Key messages

- We estimated the long-term effects of years of exposure to cash transfer programmes during childhood and adolescence (ages 0–17 years) on depression in early adulthood (18–30 years) using a quasi-experimental approach that exploits variation across three countries (Colombia, Mexico, and South Africa).
- Each additional year of exposure to cash transfer programmes during childhood was associated with a 4% reduction in the odds of depressive symptoms in early adulthood.
- Although the effect size is small, the fact that a population effect arises is noteworthy given that only a fraction of all children received the transfer.
- We did not find any effect of years of exposure to cash transfer programmes on secondary school completion, and we found a negative effect on employment in early adulthood.

## Introduction

Over 30% of the world’s young people live in poverty and face multiple deprivations including inadequate living standards, poor health, lack of education, poor quality of work, and violence (Alkire et al. 2019). At the same time, 10–20% of children and adolescents worldwide experience mental health problems, which impact long-term educational, employment, and economic outcomes, thereby increasing the risk of persistent poverty (Kieling et al. 2011). Extensive research suggests that poverty is associated with mental illness among adolescents and young adults (Lund et al. 2010, Sayeed and Fernando 2018, Ridley et al. 2020, Marchi et al. 2024). Although mechanisms underlying this relationship are not well understood (Ridley et al. 2020, Evans-Lacko et al. 2023), studies suggest that a vicious cycle between poverty and mental health starts early in life and deepens as children become young adults (Collis et al. 2009). Mental illness during childhood may influence the risk of poverty in early adulthood, by hampering the ability to succeed in education and employment (Clayborne et al. 2018). In turn, poverty during childhood may increase exposure to risk factors for mental illness, such as financial distress, violence or intrahousehold conflict (Evans and Kim 2007, Ridley et al. 2020).

Since the late 1990s, many low- and middle-income countries (LMICs) have implemented nationwide cash transfer (CT) programmes to address poverty. These programmes directly transfer income to poor households, but they may vary in structure. Some are conditional (they tie cash payments to parents’ human capital investments in their children, such as school attendance and participation in vaccination and other public health programmes), while others provide support without such requirements, referred to as unconditional CTs (Lagarde et al. 2007, Baird et al. 2014, Owusu-Addo et al. 2018). In this study, we focus on three countries with large national CT programmes introduced in the 1990s and early 2000s: Brazil, Mexico, and South Africa.

Brazil’s Bolsa Família, launched in 2003, has grown to become one of the largest conditional CT programmes in the world, reaching ∼13.8 million families or ∼25% of the country’s population. Similarly, Mexico’s Oportunidades, also conditional, established in 1997, was expanded over the years to cover >5 million families, with a budget of $3.7 billion by 2007, reflecting its significant role in the country’s social protection strategy. South Africa’s Child Support Grant (CSG), initiated in 1998, is an unconditional programme that targets primary caregivers to improve food security and overall child well-being. By 2020, 12.5 million children, or 63% of South Africa’s child population, were receiving this grant, illustrating its widespread reach and impact (see the Supplementary material for more details on programmes).

While the three programmes differ in a number of aspects, they also share several important common features, including a focus on CTs as a key poverty reduction strategy, a goal to improve family and child outcomes, and the targeting of very low-income households through means testing. These are unprecedented programmes representing significant investments on CTs in each of the countries, which have been shown to have improved the economic well-being of households (Angelucci et al. 2012, Bastagli et al. 2019). While South Africa’s child grant programme did not involve conditionality, both Mexico’s and Colombia’s programmes incorporated conditions for the transfer that were very similar, i.e. children’s school attendance and health check-ups.

Extensive research has examined the impact of CTs on children’s health, nutrition, and education (Lagarde et al. 2007, Baird et al. 2014, Cooper et al. 2020), but few studies have examined their impact on youth mental health and few look at long-term effects. Two recent reviews focused on the impact of CTs on youth mental health (Zimmerman et al. 2021, Zaneva et al. 2022). A total of 18 studies were identified, although the maximum follow-up period was 5 years. The majority of the identified studies suggested a positive short-term association between CTs and mental health outcomes, in particular reduced symptoms of depression and anxiety; however, the reviews also highlight the variability of these effects, with some studies reporting no impacts (Zimmerman et al. 2021, Zaneva et al. 2022).

There are several pathways by which earlier—and therefore longer-term—exposure to CTs may lead to sustained benefits in mental health into early adulthood. First, higher household income may reduce financial insecurity during childhood, a well-known risk factor for later mental health problems (Ohrnberger et al. 2020). Higher income may increase household stability, which would in turn reduce household conflict and distress, potentially improving both parental and child mental health and parent–child relationships. Reducing the risk of mental disorders among parents (Ozer et al. 2011, Powell-Jackson et al. 2016, Eyal and Burns, 2019) may also translate into better long-term mental health outcomes for children and adolescents. A key goal of CTs is to increase school attendance, which may improve young people’s employment opportunities and mental health. Education and employment are two key mechanisms by which CTs may affect mental health (Lund et al. 2018). However, the impacts of CTs on these outcomes are likely complex. CTs may increase primary school completion and thus improve mental health during adolescence, which may increase secondary school completion rates and improve mental health in early adulthood. The impact of CTs on employment in early adulthood is less clear: on the one hand, better mental health and more human capital in earlier periods may increase employment outcomes in early adulthood. On the other hand,, CTs may also reduce employment in early adulthood by increasing the length of post-secondary schooling, thus reducing accumulated work experience.

Research over the last decades suggests that CTs increase primary and secondary school completion (Garcia and Saavedra 2017). However, recent studies on long-term impacts of CTs during childhood on early adulthood outcomes have mixed findings. Some studies report that earlier age of starting to receive CTs—and therefore having been exposed longer to CTs—leads to better educational and labour market outcomes in early adulthood (Behrman and Hoddinott 2005, Fernald et al. 2009, Barham et al. 2013, Parker and Vogl 2018, Sanchez 2019). However, a recent review by Millán et al. (2019) concluded that while some studies find positive effects on educational attainment, results are mixed and do not yet offer conclusive evidence.

Establishing the causal impact of CTs is challenging due to selection bias: CT programmes typically target low-income, disadvantaged families, so that CT recipients are by definition more disadvantaged and have worse outcomes than non-recipients. A ‘naïve’ comparison between CT recipients and non-recipients, therefore, will provide downwardly biased estimates, even controlling for observables or using matching techniques. Some studies have used a randomized controlled trial design (Ozer et al. 2011, Attah et al. 2016, Zimmerman et al. 2021). However, these studies often have a short follow-up and face the challenge that programmes have been scaled up to the entire population, making it difficult to rely on a control group, as controls may also receive CTs because many programmes have already been scaled up in countries where the RCTs are performed (Fernald et al. 2008).

In this study, we propose a novel design to try to understand the long-term impacts of CTs on mental health that exploits variation in the timing of the introduction and expansion of CT programmes across countries and birth cohorts. In particular, we assess whether earlier—and therefore longer—cumulative years of potential exposure to a CT programme for a given country and birth cohort during childhood and adolescence (0–17 years) reduce that cohort’s later depressive symptoms. We use harmonized data from Colombia, Mexico, and South Africa and exploit variation in the cumulative years of exposure to the CT that arose from cross-country differences in the year CT programmes were introduced and birth-cohort differences in the age of children. This approach enables us to examine whether individuals who were younger when CT programmes were introduced, and consequently had greater cumulative exposure to CTs, have better mental health outcomes in young adulthood than individuals who were older when CT programmes were introduced. Rather than capturing the direct impact of providing CTs to individuals, this approach enables us to estimate the impact of the policy on the average health of children.

We hypothesize that countries and cohorts with longer cumulative years of exposure to CTs during childhood and adolescence have fewer depressive symptoms in early adulthood. While we explore potential population-wide effects of CTs, we acknowledge that these effects could be moderated by structural factors such as regional economic disparities, access to healthcare and education, and the conditionality of the programmes. To shed light on some of the potential mechanisms, we also examine how CTs during childhood and adolescence influence secondary school completion and employment in early adulthood. Given the high coverage rates of CTs in the countries we examine, we expect that CTs may have population wide effects for entire cohorts of children. However, we expect effects to be stronger for children from families below the poverty line, as they were more likely to be target recipients and more likely to fulfil eligibility. By examining these diverse settings, we aim to contribute to a broader understanding of how CT programmes can influence mental health outcomes across varied socio-economic and cultural contexts. To our knowledge, this is the first study to examine the long-term impact of cumulative exposure to CT programmes during childhood and adolescence on depressive symptoms in early adulthood.

## Materials and methods

An overview of CT programmes in each country is provided in Supplementary File S1.

### Data

This study is part of the CHANCES-6 study (Bauer et al. 2021), a study of the effect of CTs on youth mental health and life chances in six LMICs . The data sources analysed for this study represent a subset of available data sources that had publicly available data on nationally representative samples of individuals who could have been exposed to CT programmes during childhood and with information on depressive symptoms.

Data for Colombia come from the 2015 National Mental Health Survey, a nationally representative, probabilistic cross-sectional survey (Gomez-Restrepo et al. 2016). The overall sample had 14 496 participants. The survey involves in-depth questionnaires for different age groups: children (7–11 years), adolescents (12–17 years), and adults (≥18 years).

Data for South Africa came from the nationally representative National Income Dynamics Study (NIDS), a longitudinal survey that aims to provide representative socio-economic, behavioural, and anthropometric data for South Africa. This longitudinal study began in 2008 with a nationally representative sample of >28 000 individuals (Leibbrandt et al. 2009). The final sample at Wave 1 resulted in 7305 participating households (69% response rate). We used Wave 4 data, collected in 2014, from participants aged 18–30 years.

We used data from the third wave (2009–2012) of the Mexican Family Life Survey (MxFLS-3), a longitudinal, nationally representative survey of 8400 households in 150 urban and rural communities across Mexico, collected over three waves from 2002 to 2012.

Our final analytical sample included 32 883 respondents aged 18–30 years at the time of survey. Inclusion criteria are participants aged 18–30 years with available data. After excluding individuals with missing information on key variables (age, depressive symptoms or socio-economic conditions), the final sample included 15 431 individuals: 6789 from Mexico, 5802 from South Africa, and 2840 from Colombia. Table 1 shows the demographics of included study participants and those who were excluded due to missing data.

**Table 1.** Descriptive characteristics for participants with missing and without missing data^a^

	Included study participants (*N* = 15 431)	Participants with missing data (*N* = 17 441)
Potential years of exposure, years (SD)	7.96 (5.6)	7.66 (4.3)
Female, %	8511 (55%)	7803 (49%)
Age, years (SD)	23.5 (3.7)	23.9 (3.7)
Poverty (% yes)	8231(53%)	6457 (59%)
Normalized depressive symptoms (SD)	0.18 (0.19)	0.20 (0.18)
Depression binary (% over threshold for depression)	2127(13%)	245 (17%)
Schooling (% not completed secondary)	11 130 (72%)	6760 (73%)

a Participants aged 18–30 years in South Africa (NIDS Wave 4: 2014), Colombia (Encuesta Nacional de Salud Mental: 2015), and Mexico (MxFLS Round 3: 2012).

### Research design

We used a quasi-experimental design to assess the impact of duration of exposure to the programme on depressive symptoms. Rather than relying on information on whether participants received the CT throughout their childhood—which would be endogenous given that programmes target low-income families—our study design relies on cross-country and cohort variation in the age of children when the programme was first introduced. In leveraging the years of exposure to CTs across countries and cohorts, our empirical strategy seeks to capture the impact of the introduction of the policy on cohorts of children and adolescents, rather than the impact of individual receipt of CTs, which by design is correlated with poverty given the targeted nature of the programme.

**Figure 1.**
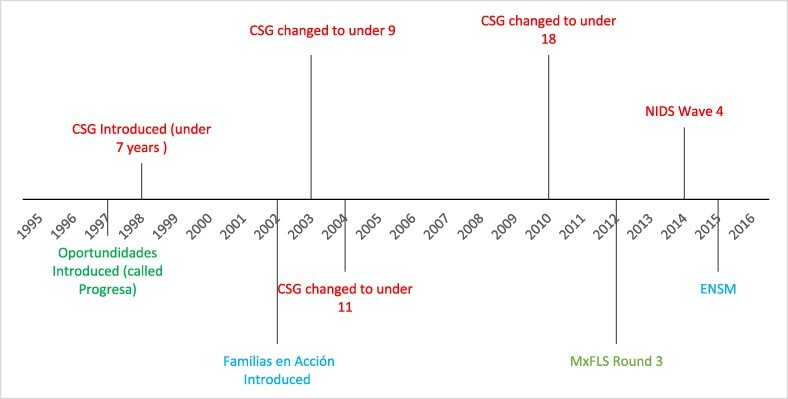
Timeline of year when CTs were introduced and when data were collected in South Africa, Mexico, and Colombia; CSG = Child Support Grant (South Africa); NIDS = National Income Dynamics Study; MxFLS = Mexico Family Life Survey; ENSM = Encuesta Nacional de Salud Mental, Colombia

CT programmes were introduced as a national policy in all three study countries at a specific moment in time. Therefore, for a given country, the age of first possible exposure to the CT is the same for all individuals born in the same year. For example, children born in 1995 in Mexico would have first been exposed to the policy at 2 years of age, as the programme was introduced in 1997. Our design exploits the fact that different countries introduced the policy in a different year (Figure 1), generating cross-country variation in the age of first exposure to the policy for cohorts born in the same year. For example, Oportunidades was introduced in 1997, so children aged 15 years in 2010 from Mexico would have been first exposed to the policy at 2 years of age. In contrast, Familias en Acción was introduced in 2002, so a child from Colombia aged 15 years in 2010 would have been first exposed to the policy at 7 years of age. Thus, participants with the same birth year vary in the age at which they were first exposed to the CT and therefore in the duration of exposure throughout their childhood.

For this study, we included all children who met country-specific age-eligibility criteria for CTs in the exposed cohort, regardless of their CT beneficiary status. However, only a fraction of these children were actual CT beneficiaries. For example, in 2015, the coverage rate of CTs was 21% in Latin America (Cecchini and Atuesta 2017). In 2016, Oportunidades in Mexico reached 6.1 million families, or 24% of all families in Mexico, Familias en Acción in Colombia reached 2.5 million families, or 17.5% of the population (Álvarez-Iglesias et al. 2021), and the CSG in South Africa reached 12.5 million families, or 63% of the child population (UNICEF 2020). A potential concern is that by examining all children born in a certain year (regardless of CT receipt status), we fail to capture small impacts that may not have changed population averages. Therefore, we also assessed the impact of years of cumulative exposure for individuals classified as poor at the time of interview. We do this by introducing an interaction between country and household poverty levels, with poverty defined using country-specific income poverty measures used to determine eligibility for the programme (further details on the measurement of poverty in each country are provided further). Because poverty status is a criterion for programme receipt, those below a country-specific poverty threshold would be more likely to have received CTs. Therefore, we expect a stronger effect of CTs among individuals below country-specific poverty thresholds.

### Measurements

#### Years of potential exposure to the CT

We calculated the maximum number of years that participants could have been exposed to CTs during childhood (from age 0 to 17 years), which was a function of date of birth, the year programme was introduced in their country of residence, the age-eligibility rules for each programme, and year of interview. For example, in Mexico, where the programme was introduced in 1997, the maximum exposure was 15 years for participants born in 1997 and interviewed at 15 years of age in 2012. The length of exposure took into account the age eligibility for each country. In Mexico and Colombia, families with children under age 18 years were eligible for CTs; hence, we counted the total number of years up to age 17 years. However, in South Africa, we accounted for the changes in age eligibility over time. The CSG was initially available only for households with children under 7 years, but this was later expanded gradually to include children up to age 18 years (Figure 1).

#### Outcome—depressive symptoms

Our primary outcome was normalized depressive symptom scores, taken from self-report questionnaires. In Colombia, depression symptoms were measured using the Self-Reporting Questionnaire (SRQ-20; Harding et al. 1980), which asks participants whether they currently have any of a list of 13 symptoms. Items are added together resulting in a total score ranging from 0 to 20, with higher scores indicating greater likelihood of depression. Individuals were categorized into either low (SRQ-20 <4) or elevated (SRQ-20 ≥4) symptomatology groups, based on previously established cut-off points in a Colombian sample (Gomez-Restrepo et al. 2016).

In South Africa, depressive symptoms were measured using the 10-item Centre for Epidemiologic Studies Depression Scale (CESD-10; Andresen et al. 1994), which asks participants to report the frequency of 10 symptoms in a four-point Likert during the past week. A cut-off of 12 on the CES-D-10 presented the most balanced sensitivity (71.4%) and specificity (72.6%) when validated against the Mini Neuropsychiatric Interview (MINI) as the gold standard comparison (Baron et al. 2017). Symptom scores were summed and categorized into either low (CES‐D <12) or elevated (CES‐D ≥12) symptomatology groups.

The Mexican Family Life Survey used the Clinical Questionnaire for the Diagnosis of Depressive Syndrome [Cuestionario Clínico para el Diagnóstico del Síndrome Depresivo (CCDSD); Calderón-Narváez 1997], which asked participants about the frequency of 21 symptoms in the last 4 weeks using a four-point scale. The total score ranged from 20 to 84 with a higher score indicating more depressive symptoms. We used previously established cut-offs (Calderón-Narváez 1997), where a score of >65 reflects depression.

Given that each scale had a different score, we estimated normalized values for each of the three scales to enable comparable units across measures. We scaled depression scores to values between 0 and 1 by using the following formula:


$${z_i} = \frac{{{x_i} - \min (x)}}{{\max (x) - \min (x)}} \\[14pt]$$

We created a cut-off for significant depression symptoms (yes/no) based on the respective binary cut-offs for each scale using validated thresholds for young people in the respective countries. Sensitivity analyses explored whether altering these cut-offs influenced the results.

#### Education and employment outcomes

Surveys include data on the highest level of schooling achieved. Participants were classified into binary groups based on whether they completed secondary schooling (1) or not (0). Employment at the time of the survey was measured as a binary variable (yes/no) using questions from each survey on whether participants were employed in the last 12 months.

#### Household poverty

For each dataset, we calculated the poverty cut-off based on socio-economic eligibility criteria for individual CT programmes. For Colombia, eligibility is determined by the System for Identifying and Selecting Beneficiaries (SISBEN), which divides households into categories, based on their estimated level of income. We followed the governmental cut-offs, in which households eligible for the Families en Acción programme must belong to the lowest of these categories, SISBEN level one. Those in the lowest SISBEN level were categorized as extreme poverty (1) and others were categorized as not in extreme poverty (0).

For South Africa, we used national poverty lines, based on the cost-of-basic-needs approach. The lower bound poverty line is calculated from the food poverty line—the amount of money an individual will need to afford the minimum required daily energy intake (often referred to as the ‘extreme’ poverty line)—plus the average amount derived from non-food items of households where the total expenditure is equal to the food poverty line (STATSSA 2019). For Wave 4, households were categorized as below the poverty line if their monthly household income was below R613 pp/m, which was the national poverty line for that year.

For Mexico, Oportunidades-Progresa eligibility is determined according to the household’s per capita income using SUP (the Spanish acronym for ‘Sistema Unico de Puntajes’). SUP scores are estimated using economic poverty as dependent variable, discriminant analysis, and a single source of national information. In our data, household incomes were compared to the ‘Canasta Normativa Alimentaria’, the national food basket, to determine poverty and eligibility. Then, a discriminant analysis was performed to create a score for each household according to its deprivations. This score was then compared with SUP scores. The threshold of 0.69 was used to classify households as being in high poverty (Pfutze 2019).

#### Empirical approach

We started by examining the association between years of exposure and outcomes using non-parametric locally weighted scatterplot smoothing (LOWESS) curves. We then implemented parametric country fixed-effects models with the following basic OLS specification:


$$\begin{aligned} {y_{\mathrm{i}}} =& \, {\beta _0} + {\beta _1}\left[ {Length{\ }of{\ }Exposure} \right]{\ } + {\beta _2}\left[ {Sex} \right]{\ } \\ &+ {\beta _3}{\left[ {Poverty{\ }Status} \right]_{}}+ {\beta _4}\left[ {3 - year{\ }birth{\ }categories} \right]{\ } \\ &+ {\beta _5}\left[ {Country} \right]{\ } + \in \end{aligned}$$

where *y* is the normalized depressive symptom score at ages 18–30 years for an individual (or a binary variable for high depressive symptomatology), secondary school completion or employment. Education and employment are binary variables. For binary outcomes, we implemented the same model but using logistic regression. From logistic regressions, we reported the marginal effects (Norton and Dowd 2018). Independent variables were the length of exposure to the CT measured in years, sex, household poverty status, 3-year birth categories, and country. Country fixed effects control for any unobserved differences between countries, while birth-cohort fixed effects control for any differences between birth cohorts common to all countries. In this model, the impact of years of exposure to the policy is identified out of variation in years of exposure within a given country, relative to the same variation in other countries, controlling for overall birth-cohort effects. An identifying assumption is that birth-cohort effects are constant across countries and are well captured by 3-year birth-cohort dummies. We discuss these assumptions in more detail later in the paper. In sensitivity analysis, we tested whether the specification of birth year impacts the results by repeating the analysis using birth year as a continuous variable.

We assessed whether the impact of CTs differed by sex by including an interaction term between years of exposure and sex. We also assessed whether the impact of CTs was greater for households classified as poor by introducing an interaction term between years of exposure and household poverty. We applied calibrated post-stratification sampling weights to adjust for disproportionate representation of sample sizes, with estimated clustered standard errors, clustered at the country level. Data were analysed using Stata 17 (StataCorp 2017).

#### Patient and public involvement

Patients and the public were not involved in this research study.

## Results

Socio-demographic characteristics of participants are reported in Table 2. The prevalence of poverty varied from 23% in Mexico to 36% in South Africa and 56% in Colombia. Depressive symptomatology was higher in Mexico (17%) than in South Africa (12%) and Colombia (9%).

**Table 2.** Descriptive data for participants aged 18–30 years in South Africa (NIDS Wave 4: 2014), Colombia (Encuesta Nacional de Salud Mental: 2015), and Mexico (MxFLS round 3: 2012)

	South Africa (*N* = 5802)	Mexico (*N* = 6789)	Colombia (*N* = 2840)
Potential years of exposure, years (SD)	6.1 (7.0)	10.1 (3.5)	6.8 (3.7)
% Female	3040 (53%)	3738 (55%)	1733 (61%)
Age, years (SD)	23.5 (3.7)	23.0 (3.5)	24.2 (3.7)
Poverty (% yes)	2067 (36%)	4461 (67%)	1589 (56%)
Normalized depressive symptoms (SD)	0.21 (0.14)	0.20 (0.23)	0.07 (0.4)
Depression binary (% over threshold for depression)	665 (12%)	1175 (17%)	337 (9%)
Schooling (% not completed secondary)	3334 (59%)	5416 (57%)	474 (17%)

**Figure 2.**
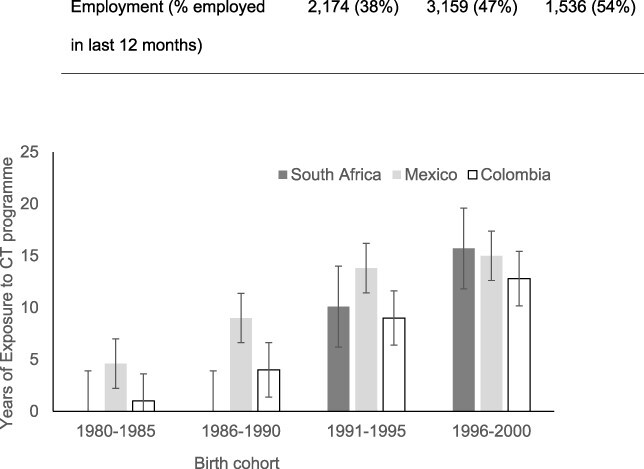
Mean years of potential exposure to the CT against birth year categories for the separate countries and harmonized dataset

To illustrate the variation in our main exposure variable, Figure 2 shows the mean years of exposure to the local CT programme by country and 3-year birth cohorts. Although there was a high correlation between years of exposure and birth year in the full sample (*r* = 0.76, *P* < 0.05), within each birth cohort, there was variation in the years of exposure between countries. For example, the cohort born in 1980–83 had a mean of 4.6 years of exposure to the CT programme in Mexico, while the same cohort had only 1 year of exposure in Colombia, and zero in South Africa. Variations in years of exposure were present across all birth cohorts.

### Years of exposure to the CT and depressive symptoms in early adulthood

**Figure 3.**
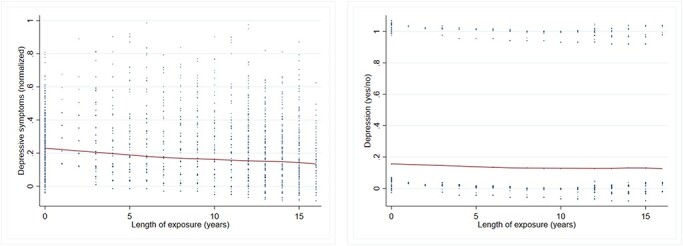
Non-parametric LOWESS curves showing the association between years of exposure and continuous depressive symptom score, controlling for 3-year birth cohort, country, sex, and poverty status

We start by examining the association between years of exposure and outcomes using non-parametric LOWESS function curves, controlling for 3-year birth cohort, country, sex, and poverty status. Figure 3 shows that as years of exposure to a CT increase, depressive symptom score declines. That is, each additional year of exposure to a CT programme from age 0 to 17 years conferred a reduction in depressive symptom scores at ages 18–30 years.

Table 3 shows linear regression coefficients for Models 1 and 2 on continuous depressive symptom scores and logistic regression coefficients for Models 3 and 4 on dichotomous depression. Each column shows a single model. Results from Model 1 suggest that each additional year of exposure to the CT is associated with 0.002 of a standard deviation reduction in normalized depressive symptom scores (*β* = −0.003, 95% confidence intervals (CIs): −0.004 to −0.01, *P* < 0.001; see Table 3, Model 1). Mean years of potential exposure to a CT was 9.5 (Table 1); this implies that children who were potentially exposed to a CT programme for 9.5 years experienced a reduction of 0.03 points on the depressive symptoms score, relative to those unexposed during childhood and adolescence. This is a relatively small effect (Cohen’s *d* = 0.02).

**Table 3.** Association between potential years of exposure to the CT and depressive symptoms among participants aged 18–30 years in Mexico, South Africa, and Colombia (*N* = 15 431)

	Model 1: continuous normalized depressive symptoms	Model 2. categorical depression (yes/no)	Model 3. interaction with sex (continuous normalized depressive symptoms)	Model 4: interaction with sex (categorical depression, yes/no)
	*β* (95% CIs)	OR (95% CIs)	*β* (95% CIs)	OR (95% CIs)
Years of potential exposure	−0.003 (−0.004, −0.001)	0.96 (0.93, 0.98)	−0.04 (−0.01, −0.002)	0.94 (0.92, 0.97)
Sex (ref.: male)	0.04 (0.04, 0.05)	1.74 (1.58, 1.92)	0.03 (0.02, 0.04)	1.66 (1.0, 1.96)
Years of potential exposure × sex (ref.: male)	N/A	N/A	0.002 (0.001, 0.003)	1.02 (0.99, 1.03)
Poverty (ref.: no poverty)	0.01 (0.01, 0.02)	1.15 (1.04, 1.26)	0.02 (0.01, 0.02)	1.15 (1.04, 1.27)
Birth year 1986–88 (ref.: 1983–85)	0.002 (−0.02, 0.03)	0.99 (0.70, 1.40)	0.003 (−0.02, 0.03)	0.99 (0.70, 1.50)
Birth year 1989–91 (ref.: 1983–85)	0.01 (−0.02,0.03)	1.11 (0.79, 1.57)	0.01 (−0.02, 0.03)	1.11 (0.79, 1.57)
Birth year 1992–94 (ref.: 1983–85)	0.01 (−0.02, 0.03)	1.711.14 (0.79, 1.63)	0.01 (−0.02, 0.03)	1.13 (0.79, 1.62)
Birth year 1995–97 (ref.: 1983–85)	0.03 (0.001, 0.06)	1.69 (1.11, 2.58)	0.03 (−0.001, 0.06)	1.68 (1.10, 2.56)
Birth year 1998–2000 (ref.: 1983–85)	0.03 (−0.003, 0.07)	1.97 (1.17, 3.34)	0.03 (−0.04, 0.06)	1.94 (1.15, 3.29)
South Africa (ref.: Mexico)	0.01 (−0.01, 0.02)	0.50 (0.42, 0.60)	0.01 (−0.01, 0.02)	0.50 (0.42, 0.60)
Colombia (ref.: Mexico)	−0.14 (−0.15, −0.13)	0.36 (0.30, 0.44)	−0.14 (−0.15, −0.13)	0.37 (0.30, 0.45)

To get a sense of the magnitude of this effect in terms of serious depressive symptomatology, Model 2 shows results using scale-specific cut-off points to define a categorical measure of depressive symptoms (yes/no), where each column shows a single model. Results suggest that each additional year of potential exposure to a CT is associated with a 4% reduction in the odds of depressive symptomatology [odds ratio (OR) = 0.96, 95% CIs: 0.93, 0.98; see Table 3, Model 3).

There was evidence of a positive sex interaction (*β* = 0.002, 95% CIs: 0.001, 0.003; see Table 3, Model 3), so the association between years of potential exposure and depressive symptom scores was stronger for females.

### Years of exposure to the CT and schooling

Table 4 shows logistic regression coefficients for Model 1 on schooling (completed secondary or not). Each column shows a single model. Results from Model 1 suggest that there is no association between years of CT exposure during childhood and secondary school completing in young adulthood, once country and birth cohorts are controlled for (OR = 1.01, 95% CIs: 0.99, 1.03; see Table 4, Model 1). The average marginal effect is 0.01 (95% CIs: 0.002, 0.02). There was no significant sex interaction (OR = 0.99, 95% CIs: 0.97, 1.00; see Table 4, Model 2); however, the effect on secondary schooling completion was positive among males (OR = 1.02, 95% CIs: 1.00, 1.04).

**Table 4.** Association between potential years of exposure to the CT and schooling (completed secondary or not) among participants aged 18–30 years in Mexico, South Africa, and Colombia (*N* = 15 431)

	Model 1: schooling (completed secondary or not)	Model 2: schooling (completed secondary or not; interaction with sex)
	Marginal effect (95% CIs)	Marginal effect (95% CIs)
Years of potential exposure	0.01 (−0.001, 0.02)	1.02 (1.00, 1.04)
Sex (ref.: male)	−0.03 (−0.04, −0.01)	0.85 (0.76, 0.97)
Years of potential exposure × sex (ref.: male)	N/A	0.99 (0.97, 1.00)
Poverty (ref.: no poverty)	0.22 (0.21, 0.23)	2.23 (2.06, 2.41)
Birth year 1986–88 (ref.: 1983–85)	−0.05 (−0.09, −0.002)	0.78 (0.55, 1.10)
Birth year 1989–91 (ref.: 1983–85)	−0.12 (−0.18, −0.06)	0.74 (0.52, 1.04)
Birth year 1992–94 (ref.: 1983–85)	−0.16 (−0.23, −0.09)	0.68 (0.48, 0.96)
Birth year 1995–97 (ref.: 1983–85)	−0.19 (−0.28, −0.09)	0.82 (0.55, 1.23)
Birth year 1998–2000 (ref.: 1983–85)	−0.04 (−0.16, 0.09)	1.18 (1.37, 3.49)
South Africa (ref.: Mexico)	0.45 (0.42, 0.48)	0.37 (0.32, 0.43)
Colombia (ref.: Mexico)	0.65 (0.61, 0.69)	1.34 (1.15, 1.55)

Model 1: marginal effects of the association between years of potential exposure and schooling (completed secondary or not).

Model 2: the interaction between sex and years of potential exposure and schooling (completed secondary school = 1).

### Years of exposure to the CT and employment on early adulthood

Findings suggest that the probability of employment decreased as years of exposure to a CT increased. That is, each additional year of exposure to a CT at ages 0–17 years was associated with a reduced probability of employment at ages 18–30 years.

Table 5 shows logistic regression coefficients for Model 1 on employment (employed in last year or not). Each column shows coefficients for a single model. Results from Model 1 suggest a negative association between length of exposure and employment (OR = 0.90, 95% CIs: 0.88, 0.91; see Table 5, Model 1); roughly, this means that each additional year of exposure to the CT programme reduced the odds of employment at ages 18–30 years by 11%. The average marginal effect is −0.04, 95% CIs: −0.05 to −0.04. Results were similar when this analysis was repeated with a subgroup of those not currently in education (Supplementary Table S2). There was no significant sex interaction (OR = 0.99, 95% CIs: 0.99, 1.01, see Table 5, Model 2).

**Table 5.** Association between potential years of exposure to the CT and employment (employed in the last year or not) among participants aged 18–30 years in Mexico, South Africa, and Colombia (*N* = 15 431)

	Model 1: Employed in the last year or not	Model 2: employed in the last year or not (interaction with sex)
	OR (95% CIs)	OR (95% CIs)
Years of potential exposure	0.90 (0.88, 0.91)	0.90 (0.89, 0.92)
Sex (ref.: male)	0.25 (0.24, 0.27)	0.28 (0.24, 0.31)
Years of potential exposure × sex (ref.: male)	N/A	0.99 (0.97, 1.00)
Poverty (ref.: no poverty)	0.50 (0.46, 0.54)	0.50 (0.46, 0.54)
Birth year 1986–88 (ref.: 1983–85)	1.07 (0.79, 1.44)	1.07 (0.80, 1.44)
Birth year 1989–91 (ref.: 1983–85)	1.10 (0.82, 1.48)	1.11 (0.83, 1.50)
Birth year 1992–94 (ref.: 1983–85)	0.96 (0.71, 1.30)	0.97 (0.72, 1.32)
Birth year 1995–97 (ref.: 1983–85)	0.96 (0.67, 1.36)	0.97 (0.68, 1.38)
Birth year 1998–2000 (ref.: 1983–85)	0.50 (0.33, 0.77)	0.51 (0.34, 0.78)
South Africa (ref.: Mexico)	0.34 (0.30, 0.40)	0.34 (0.29, 0.39)
Colombia (ref.: Mexico)	1.14 (0.99, 1.30)	1.23 (0.98, 1.29)

Model 1: the association between years of potential exposure and employment (employed in the last year or not).

Model 2: the interaction between sex and years of potential exposure and employment (employed in the last year or not).

### Sensitivity analysis

We tested the interaction between poverty and years of exposure in linear and logistic regressions for normalized depressive symptom scores and dichotomous depression categories, as well as schooling and employment (Supplementary Table S1). There was no significant interaction for either the normalized variable (*β* = 0.0004, 95% CIs: −0.001 to 0.001) or dichotomous outcome (OR = 0.99, 95% CIs: 0.97, 1.01), suggesting that the impact did not differ by poverty level. However, there was a significant interaction for schooling (OR = 0.90, 95% CIs: 0.87, 0.92), suggesting that exposure had a stronger negative effect on schooling for young people living in poverty than those not living in poverty. There was also a significant interaction for employment (OR = 1.09, 95% CIs: 1.08, 1.11), suggesting that exposure had a weaker negative effect on employment for young people living in poverty than those not living in poverty.

## Discussion

This is the first cross-country study to estimate the long-term, cumulative effects of the number of years of exposure to CTs during childhood and adolescence on depressive symptoms in early adulthood. Using harmonized data from Colombia, Mexico, and South Africa, we found a negative association between potential years of exposure and depressive symptoms, indicating that longer exposure to CTs was associated with lower depressive symptom scores—or put more simply, greater exposure led to improved mental health. We found no association with school completion and a negative association with employment, indicating that longer exposure to CTs reduced the likelihood of being employed in the last 12 months. We did not find any significant interactions between poverty and potential years of exposure on depressive symptoms.

Our findings suggest that growing up during a CT expansion may be associated with small impacts on depressive symptoms in early adulthood. While the effect sizes were small, the fact that a population effect arises is noteworthy given that only a fraction (17%, 24%, and 63% for Colombia, Mexico, and South Africa, respectively) of all children received the transfer. CT exposure could influence later mental health through multiple potential mechanisms (Evans-Lacko et al. 2023). Earlier and longer exposure to CTs may reduce financial and psychological distress at the individual and household levels. At the individual level, longer exposure to CTs may reduce financial strain by improving financial circumstances, such as increasing income or reducing debts. Reduced financial strain may also reduce the opportunity cost of time for mental health promotion activities (Ohrnberger et al. 2020), such as physical exercise or socializing with friends. Additionally, longer exposure to CTs may increase access to healthcare (Salinas-Rodríguez and Manrique-Espinoza 2013, Shei 2013, Parker and Vogl 2018), which may lead to reduced depressive symptoms. At the household level, longer exposure to CTs may reduce uncertainty and anxiety about future income. A reduction in the risk of common mental disorders among parents (Ozer et al. 2011, Powell-Jackson et al. 2016, Eyal and Burns 2019) may also translate into positive long-term mental health outcomes for children and adolescents.

Our results do not fully support the hypothesis that improvements in school completion may have contributed to reduced depressive symptoms, as we did not find an effect on schooling, although there was some suggestion that this effect may have been positive for men. It is also surprising that we find a negative effect on employment. One possible interpretation is that longer exposure to CTs may reduce the need for employment in young adulthood, which may in turn reduce the risk of depressive symptoms. Future research should seek to clarify the mechanisms by which the length of exposure to CTs improves depressive symptoms.

Our findings may reflect the effects of receiving CTs at a younger age rather than simply longer exposure. Although we controlled for birth year, participants exposed to the CT for longer also tended to receive the intervention earlier in childhood. Studies have shown that receiving CTs earlier in life has greater impacts on health, development, and education (Behrman and Hoddinott 2005, Fernald et al. 2009, Barham et al. 2013). Early life investment theory states that the earlier the intervention, the greater the human capital return (Heckman 2012). Developmental neuroscience also suggests that the brain is more plastic in early life, implying that interventions may have stronger effects if they begin earlier (Knudsen et al. 2006, Cicchetti 2015). Early intervention may prevent the development of pathology, thus reducing risk factors and increasing protective factors for long-term mental health (Cunha and Heckman 2016, Patel et al. 2018, Knapp and Wong 2020). Consistently, research suggests that early intervention leads to greater improvements in mental health (Membride 2016, Mcgorry and Mei 2018).

### Strengths and limitations

This is the first study to assess the long-term, cumulative impacts of the length of exposure to CTs on young adults’ depressive symptoms and their social determinants. By using a harmonized dataset across three countries with programmes implemented at varied times, we were able to examine the impact of length of exposure to CTs on outcomes. However, this approach has limitations associated with comparability of measures of depressive symptoms and poverty across countries. We normalized the depressive symptom scores for statistical comparison, but we cannot be sure that the different measures are fully comparable.

A second limitation is that our study does not directly assess the impact of receiving CTs, but it estimates whether cohorts of children who grew up during programme expansion had better mental health outcomes in early adulthood. Because at least half of the population in our study did not receive the CT, it is likely that our study offers ‘lower bound’ estimates of the programme impact, which should be larger for the children receiving CTs. Additionally, while our fixed-effects models help control for time-invariant factors, and birth-cohort fixed effects control for common trends affecting all children in all countries, we acknowledge that time-varying structural changes, such as shifts in regional economic conditions or healthcare access, could introduce confounding bias. Future research should consider more granular analyses that account for these structural changes. Moreover, the effects of CTs may be influenced by broader socio-political and economic dynamics, which can either enhance or limit their impact. As highlighted by Mathias et al. (2024) and Burgess (2024), these dynamics underscore the need to situate mental health improvement approaches within the broader political landscape. While CT programmes are a critical component of social interventions, their ability to produce wide-ranging and sustained impacts may be constrained in environments where structural barriers remain unaddressed. This perspective aligns with the socio-political economy approach, which stresses the importance of considering the wider context in which these programmes are implemented.

In sensitivity analyses, we could not confirm that the effect of years of exposure on mental health was stronger for children from poor households at the time of interview. There are several potential explanations for this discrepancy. First, poverty was not measured at the time participants received the CT, but rather at the time of data collection, once families had received the CT for many years. Poverty status may have changed for many families, partly because of receiving the CT; hence, our poverty measure may not reflect their poverty status when the CT was introduced. A second explanation may be that the policy did not only benefit children classified as poor, but that there are wider, population-level benefits to the mental health of children arising from the introduction of CT programmes. This may occur, for example, if CT programmes reduce crime or social unrest, increase social capital and cohesion, in a way that benefits all children. While our data are not well suited to explore this hypothesis, future studies could examine this possibility. However, it may be the case that, in addition to direct effects, our study captures the indirect effect of CTs on non-beneficiaries. CTs tend to be pooled within families and communities and can have positive general, population-level impacts on regional economies (Egger et al. 2022). It could be seen as a strength of our analysis that we are examining population-level effects of CTs.

Finally, this analysis assumes that years of exposure to CT effects on mental health are constant across countries. It may be that an extra year of exposure to the South African CT programme, for example, confers different benefits for depressive symptoms compared to the Colombian CT programme. While exploring cross-country differences is not possible in these analyses—as harmonization across countries with CT programmes that start at different times allows us to manipulate variation in the length of exposure—future research should explore how the length of exposure to CTs differentially impacts youth depressive symptoms in separate countries with different CT programmes.

## Conclusion

Using data from three countries, we find that longer years of exposure to CTs during childhood and adolescence are associated with a small but significant reduction in depressive symptoms in early adulthood. These findings have important implications for policy and suggest that CTs introduced at an earlier age and for as long as possible may yield mental health benefits later in life. However, it is crucial to recognize that these effects are deeply intertwined with broader structural factors. Further research is required to examine the causal mechanisms that may explain these effects and to better understand how socio-political and economic dynamics shape the effectiveness of CT programmes.

## Supplementary Material

czae102_Supp

## Data Availability

Data are available upon reasonable request.

## Reflexivity statement

The authors of the study include a lead female early-career researcher, a senior author, and a majority of female authors spanning multiple levels of seniority, and diversity of disciplinary expertise and geographic location, including the majority of authors being based in LMICs.

## References

[R1] Alkire S, Kanagaratnam U, Suppa N. The Global Multidimen-sional Poverty Index (MPI) 2019. OPHI Methodological Note 47. Oxford: Oxford Poverty and Human Development Initiative, 2019.

[R2] A´lvarez-iglesias A, Hessel P, Bauer A et al. Extending COVID-Related Reforms to Conditional Cash Transfers Could Improve the Life Chances of Young People in Colom-bia. blogs.lse. 2021. https://blogs.lse.ac.uk/latamcaribbean/2021/05/10/extending-covid-related-reforms-to-conditional-cash-transfers-could-improve-the-life-chances-of-young-people-in-colombia/ (20 April 2024, date last accessed).

[R3] Andresen EM, Malmgren JA, Carter WB et al. Screening for depression in well older adults: evaluation of a short form of the CES-D. *Am J Prevent Med* 1994;10:77–84. doi: 10.1016/S0749-3797(18)30622-6 8037935

[R4] Angelucci M, Attanasio O, Di Maro V. The impact of Oportu-nidades on consumption, savings and transfers. *Fiscal Stud* 2012;33:305–34. doi: 10.1111/j.1475-5890.2012.00163.x

[R5] Attah R, Barca V, Kardan A et al. Can social protection affect psychosocial wellbeing and why does this matter? Lessons from cash transfers in sub-Saharan Africa. *J Dev Stud* 2016;52:1115–31. doi: 10.1080/00220388.2015.1134777

[R6] Baird S, Ferreira F, Özler B et al. Conditional, uncon-ditional and everything in between: a systematic review of the effects of cash transfer programs on schooling outcomes. *J Dev Effective* 2014;6:1–43. doi: 10.1080/19439342.2014.890362

[R7] Barham T, Macours K, Maluccio JA. Boys’ cognitive skill for-mation and physical growth: long-term experimental evidence on critical ages for early childhood interventions. *Am Econ Rev* 2013;10:467–71. doi: 10.1257/aer.103.3.467 29524932

[R8] Baron EC, Davies T, Lund C. Validation of the 10-item Centre for Epidemiological Studies Depression Scale (CES-D-10) in Zulu, Xhosa and Afrikaans populations in South Africa. *BMC Psychiatr* 2017;17:6. doi: 10.1186/s12888-016-1178-x PMC5223549 28068955

[R9] Bastagli F, Hagen-Zanker J, Harman L et al. The impact of cash transfers: a review of the evidence from low- and middle-income countries. *J Soc Policy* 2019;48:569–94. doi: 10.1017/S0047279418000715

[R10] Bauer A, Baltra RA, Pabon MA et al. Examining the dynamics between young people’s mental health, poverty and life chances in six low- and middle-income countries: protocol for the CHANCES-6 study. *Social Psychiatry Psychiatr Epidemiology* 2021;56:1687–703. doi: 10.1007/s00127-021-02043-7 PMC8286885 34279693

[R11] Behrman JR, Hoddinott J. Programme evaluation with unob-served heterogeneity and selective implementation: the Mexican PROGRESA impact on child nutrition. *Oxf Bull Econ Stat* 2005;67:547–69. doi: 10.1111/j.1468-0084.2005.00131.x

[R12] Burgess RA . The struggle for the social: rejecting the false sepa-ration of ‘social’ worlds in mental health spaces. *Soc Psychiatry Psychiatr Epidemiol* 2024;59:409–16. doi: 10.1007/s00127-023-02510-3 37400665 PMC10944383

[R13] Calderón-Narváez G . 1997. Un cuestionario para simplificar el diagnóstico del síndrome depresivo. *Revista de Neuro-psiquiatría* 6: 127–35.

[R14] Cecchini S, Atuesta B. Conditional cash transfer programmes in Latin America and the Caribbean: coverage and investment trends. Available at SSRN 3037640, 2017.

[R15] Cicchetti D . Neural plasticity, sensitive periods, and psy-chopathology. *Dev Psychopathol* 2015;27:319–20. doi: 10.1017/S0954579415000012 25997757

[R16] Clayborne ZM, Varin M, Colman I. Adolescent depression and long-term psychosocial outcomes: a systematic review and meta-analysis. *J Am Acad Child Adolesc Psychiatry* 2018;58:72–79. doi: 10.1016/j.jaac.2018.07.896 30577941

[R17] Collis G, Hackett P, Hotopf M et al. Childhood mental health and life chances in post-war Britain. 2009.

[R18] Cooper JE, Benmarhnia T, Koski A et al. Cash transfer pro-grams have differential effects on health: a review of the literature from low and middle-income countries. *Soc Sci Med* 2020;247:112806. doi: 10.1016/j.socscimed.2020.112806 32086171

[R19] Cunha F, Heckman J. Decomposing trends in inequality in earn-ings into forecastable and uncertain components. *J Labor Econ* 2016;34:S31–65. doi: 10.1086/684121 27087741 PMC4827721

[R20] Egger D, Haushofer J, Miguel E et al. General equilibrium effects of cash transfers: experimental evidence from Kenya. *Econometrica* 2022;90:2603–43. doi: 10.3982/ECTA17945

[R21] Evans GW, Kim P. Childhood poverty and health: cumulative risk exposure and stress dysregulation. *Psychol Sci* 2007;18:953–57. doi: 10.1111/j.1467-9280.2007.02008.x 17958708

[R22] Evans-Lacko AR, Bauer A, Garman E et al. Potential mecha-nisms by which cash transfer programmes could improve the mental health and life chances of young people: a conceptual framework and lines of enquiry for research and policy. *Global Mental Health* 2023;10:e13. doi: 10.1017/gmh.2023.4 PMC10579689 37854414

[R23] Eyal K, Burns J. The parent trap: cash transfers and the inter-generational transmission of depressive symptoms in South Africa. *World Dev* 2019;117:211–29. doi: 10.1016/j.worlddev.2019.01.014

[R24] Fernald LC, Gertler PJ, Neufeld LM. Role of cash in conditional cash transfer programmes for child health, growth, and develop-ment: an analysis of Mexico’s Oportunidades. *Lancet* 2008;371:828–37. doi: 10.1016/S0140-6736(08)60382-7 18328930 PMC2779574

[R25] Fernald LC, Gertler PJ, Neufeld LM. 10-year effect of Oportu-nidades, Mexico’s conditional cash transfer programme, on child growth, cognition, language, and behaviour: a longitudinal follow-up study. *Lancet* 2009;374:1997–2005. doi: 10.1016/S0140-6736(09)61676-7 19892392

[R26] Garcia S, Saavedra JE. Educational impacts and cost-effectiveness of conditional cash transfer programs in developing countries: a meta-analysis. *Rev Educ Res* 2017;87:921–65. doi: 10.3102/0034654317723008

[R27] Gomez-Restrepo C, De Santacruz C, Rodriguez MN et al. Colom-bia 2015 National Mental Health Survey. *Study Protocol Revista Colombiana Ds Psiquiatria* 2016;45:2–8. 10.1016/j.rcp.2016.04.007 27993252

[R28] Harding TW, Mv D, Baltazar J et al. Mental disorders in pri-mary health care: a study of their frequency and diagnosis in four developing countries. *Psychol Med* 1980;10:231–41. doi: 10.1017/S0033291700043993 7384326

[R29] Heckman J . The developmental origins of health. *Health Econ* 2012;21:24. doi: 10.1002/hec.1802 PMC3351141 22147625

[R30] Kieling C, Baker-Henningham H, Belfer M et al. Child and ado-lescent mental health worldwide: evidence for action. *Lancet* 2011;378:1515–25. doi: 10.1016/S0140-6736(11)60827-1 22008427

[R31] Knapp MW, Wong G. Economics and mental health: the current scenario. *World Psychiatry* 2020;19:3–14. doi: 10.1002/wps.20692 31922693 PMC6953559

[R32] Knudsen EI, Heckman JJ, Cameron JL et al. Economic, neurobiological, and behavioral perspectives on building America’s future workforce. *Proc Natl Acad Sci* 2006;103:10155–62. doi: 10.1073/pnas.0600888103 16801553 PMC1502427

[R33] Lagarde M, Haines A, Palmer N. Conditional cash transfers for improving uptake of health interventions in low- and middle-income countries: a systematic review. *JAMA* 2007;298:1900–10. doi: 10.1001/jama.298.16.1900 17954541

[R34] Leibbrandt M, Woolard I, Villiers LD. Methodology: Report on NIDS Wave 1. 2009.

[R35] Lund C, Breen A, Flisher AJ et al. Poverty and common mental disorders in low and middle income countries: a systematic review. *Soc Sci Med* 2010;71:517–28. doi: 10.1016/j.socscimed.2010.04.027 20621748 PMC4991761

[R36] Lund C, Brooke-Sumner C, Baingana F et al. Social determi-nants of mental disorders and the Sustainable Development Goals: a systematic review of reviews. *Lancet Psychiatry* 2018;5:357–69. doi: 10.1016/S2215-0366(18)30060-9 29580610

[R38] Marchi M, Alkema A, Xia C et al. Investigating the impact of poverty on mental illness in the UK Biobank using Mendelian randomization. *Nat Human Behav* 2024;8:1771–83. doi: 10.1038/s41562-024-01919-3 38987359 PMC11420075

[R40] Mathias K, Bunkley N, Pillai P et al. Inverting the deficit model in global mental health: an examination of strengths and assets of community mental health care in Ghana, India, Occupied Pales-tinian territories, and South Africa. *PLOS Global Public Health* 2024;4:e0002575. doi: 10.1371/journal.pgph.0002575 PMC10911620 38437223

[R41] Mcgorry PD, Mei C. Early intervention in youth mental health: progress and future directions. *BMJ Mental Health* 2018;21:182–84. 10.1136/ebmental-2018-300060 PMC10270418 30352884

[R43] Membride H . Mental health: early intervention and prevention in children and young people. *Br J Nurs* 2016;25:552–57. doi: 10.12968/bjon.2016.25.10.552 27231738

[R44] Millán TM, Barham T, Macours K et al. Long-term impacts of conditional cash transfers: review of the evidence. *World Bank Res Obs* 2019;34:119–59. doi: 10.1093/wbro/lky005

[R45] Norton ECD, Dowd BE. Log odds and the interpretation of logit models. *Health Serv Res* 2018;53:859–78. doi: 10.1111/1475-6773.12712 28560732 PMC5867187

[R46] Ohrnberger J, Fichera E, Sutton M et al. The effect of cash transfers on mental health—new evidence from South Africa. *BMC Public Health* 2020;20:1–13. doi: 10.1186/s12889-020-08596-7 32245377 PMC7118950

[R47] Owusu-Addo E, Renzaho AMN, Smith BJ. The impact of cash transfers on social determinants of health and health inequalities in sub-Saharan Africa: a systematic review. *Health Policy Plann* 2018;33:675–96. doi: 10.1093/heapol/czy020 PMC5951115 29762708

[R48] Ozer EJ, Fernald LC, Weber A et al. Does alleviating poverty affect mothers’ depressive symptoms? A quasi-experimental investigation of Mexico’s Oportunidades programme. *Int J Epidemiol* 2011;40:1565–76. doi: 10.1093/ije/dyr103 21737404 PMC3235019

[R49] Parker SW, Vogl T. Do conditional cash transfers improve eco-nomic outcomes in the next generation? Evidence from Mexico. National Bureau of Economic Research No. w24303, 2018.

[R50] Patel V, Saxena S, Lund C et al. The Lancet Commission on global mental health and sustainable development. *Lancet* 2018;392:1553–98. doi: 10.1016/S0140-6736(18)31612-X 30314863

[R51] Pfutze T . Should program graduation be better targeted? The other schooling outcomes of Mexico’s Oportunidades. *World Dev* 2019;123:104625. doi: 10.1016/j.worlddev.2019.104625

[R52] Powell-Jackson T, Pereira SK, Dutt V et al. Cash transfers, maternal depression and emotional well-being: quasi-experimental evidence from India’s Janani Suraksha Yojana programme. *Soc Sci Med* 2016;162:210–18. doi: 10.1016/j.socscimed.2016.06.034 27387651

[R53] Ridley MW, Rao G, Schilbach F et al. Poverty, depres-sion and anxiety: causal evidence and mechanisms. *Science* 2020;370:eaay0214. doi: 10.1126/science.aay0214 33303583

[R54] Salinas-Rodríguez A, Manrique-Espinoza BS. Effect of the condi-tional cash transfer program Oportunidades on vaccination cover-age in older Mexican people. *BMC Int Health Hum Rights* 2013;13:30. doi: 10.1186/1472-698X-13-30 PMC3711738 23835202

[R55] Sanchez A . Impact of the Juntos Conditional Cash Transfer Pro-gramme in Peru on Nutritional and Cognitive Outcomes: Does the Age of Exposure Matter? 2019. http://younglives.org.uk/content/impact-juntos-conditional-cash-transfer-programme-peru-nutritional-and-cognitive-outcomes (21 April 2024, date last accessed).

[R56] Sayeed S, Fernando D. Poor mental health: the relation-ship between poverty and mental health in lower and mid-dle income countries. *AMSA J Glob Health* 2018;12:15–18.

[R57] Shei A . Brazil’s conditional cash transfer program associ-ated with declines in infant mortality rates. *Health Affairs* 2013;32:1274–81. doi: 10.1377/hlthaff.2012.0827 23836744

[R58] StataCorp . *Stata Statistical Software: Release 15*. College Station, TX: StataCorp LLC, 2017.

[R59] STATSSA . National Statistics. Statistics South Africa. 2019. http://www.statssa.gov.za/publications/P03101/P031012019.pdf (21 April 2024, date last accessed).

[R60] UNICEF . Universal child benefit case studies: the experience of South Africa. Unicef, 2020. https://www.unicef.org/media/70486/file/ZAF-case-study-2020.pdf (21 April 2024, date last accessed).

[R61] Zaneva M, Guzman-Holst C, Reeves A et al. The impact of monetary poverty alleviation programs on children’s and adoles-cents’ mental health: a systematic review and meta-analysis across low-, middle-, and high-income countries. *J Adolesc Health* 2022;71:147–56. doi: 10.1016/j.jadohealth.2022.02.011 35430146

[R62] Zimmerman A, Garman E, Avendano-Pabon M et al. The impact of cash transfers on mental health in children and young people in low-income and middle-income countries: a systematic review and meta-analysis. *BMJ Global Health* 2021;6:e004661. doi: 10.1136/bmjgh-2020-004661 PMC8088245 33906845

