# Supplementary material for: The long-term effects of cash transfer programmes on young adults’ mental health: a quasi-experimental study of Colombia, Mexico, and South Africa
Source: Health Policy Plan. 2024 Nov 1;40(2):206–17. doi: 10.1093/heapol/czae102 (PMC11800976; doi:10.1093/heapol/czae102)
Supplement: czae102_Supp [file czae102_supp.zip › czae102_Supp/Revised Tables Age Exposure_Clean_With References_26 Nov 2024.docx]

**Tables**

Table 1. Descriptive characteristics for participants with missing and without missing data^1^

|  | Included study participants (N=15,431) | Participants with missing data  (N= 17,441) |
| --- | --- | --- |
| Potential years of exposure, years (SD) | 7.96 (5.6) | 7.66 (4.3) |
| % female | 8,511 (55%) | 7,803 (49%) |
| Age, years (SD) | 23.5 (3.7) | 23.9 (3.7) |
| Poverty (% yes) | 8,231(53%) | 6,457 (59%) |
| Normalised Depressive symptoms (SD) | 0.18 (0.19) | 0.20 (0.18) |
| Depression binary (% over threshold for depression) | 2,127(13%) | 245 (17%) |
| Schooling (% not completed secondary) | 11,130 (72%) | 6,760 (73%) |

^1^ Participants aged 18-30 years in South Africa (NIDS Wave 4: 2014), Colombia (ENSM: 2015) and Mexico (MxFLS round 3: 2012).

Table 2. Descriptive data for participants 18-30 years in South Africa (NIDS Wave 4: 2014), Colombia (ENSM: 2015) and Mexico (MxFLS round 3: 2012).

|  | South Africa (N=5,802) | Mexico  (N= 6,789) | Colombia  (N=2,840) |
| --- | --- | --- | --- |
| Potential years of exposure, years (SD) | 6.1 (7.0) | 10.1 (3.5) | 6.8 (3.7) |
| % female | 3,040(53%) | 3,738 (55%) | 1,733 (61%) |
| Age, years (SD) | 23.5 (3.7) | 23.0 (3.5) | 24.2 (3.7) |
| Poverty (% yes) | 2,067 (36%) | 4,461 (67%) | 1,589 (56%) |
| Normalised Depressive symptoms (SD) | 0.21 (0.14) | 0.20 (0.23) | 0.07 (0.4) |
| Depression binary (% over threshold for depression) | 665 (12%) | 1,175 (17%) | 337 (9%) |
| Schooling (% not completed secondary) | 3,334 (59%) | 5,416 (57%) | 474 (17%) |

Table 3. Association between potential years of exposure to the CT and depressive symptoms among participants aged 18-30 years from Mexico, South Africa, and Colombia (N= 15,431).

|  | Model 1: Continuous normalised depressive symptoms | Model 2. Categorical depression (yes/no) | Model 3. Interaction with sex (continuous normalised depressive symptoms) | Model 4: Interaction with sex (categorical depression, yes/no) |
| --- | --- | --- | --- | --- |
|  | B  (95% CIs) | OR  (95% CIs) | B  (95% CIs*)* | OR  (95% CIs*)* |
| Years of potential exposure | -0.003  (-0.004, -0.001) | 0.96  (0.93, 0.98) | -0.04  (-0.01, -0.002) | 0.94  (0.92, 0.97) |
| Sex (ref.: male) | 0.04  (0.04, 0.05) | 1.74  (1.58, 1.92) | 0.03  (0.02, 0.04) | 1.66  (1.0, 1.96) |
| Years of potential exposure * sex (ref.: male) | N/A | N/A | 0.002  (0.001, 0.003) | 1.02  (0.99, 1.03) |
| Poverty (ref.: no poverty) | 0.01  (0.01, 0.02) | 1.15  (1.04, 1.26) | 0.02  (0.01, 0.02) | 1.15  (1.04, 1.27) |
| Birth year 1986-1988 (ref.: 1983-1985) | 0.002  (-0.02, 0.03) | 0.99  (0.70, 1.40) | 0.003  (-0.02, 0.03) | 0.99  (0.70, 1.50) |
| Birth year 1989-1991 (ref.: 1983-1985) | 0.01  (-0.02,0.03) | 1.11  (0.79, 1.57) | 0.01  (-0.02, 0.03) | 1.11  (0.79, 1.57) |
| Birth year 1992-1994 (ref.: 1983-1985) | 0.01  (-0.02, 0.03) | 1.711.14  (0.79, 1.63) | 0.01  (-0.02, 0.03) | 1.13  (0.79, 1.62) |
| Birth year 1995-1997 (ref.: 1983-1985) | 0.03  (0.001, 0.06) | 1.69  (1.11, 2.58) | 0.03  (-0.001, 0.06) | 1.68  (1.10, 2.56) |
| Birth year 1998-2000 (ref.: 1983-1985) | 0.03  (-0.003, 0.07) | 1.97  (1.17, 3.34) | 0.03  (-0.04, 0.06) | 1.94  (1.15, 3.29) |
| South Africa (ref.: Mexico) | 0.01  (-0.01, 0.02) | 0.50  (0.42, 0.60) | 0.01  (-0.01, 0.02) | 0.50  (0.42, 0.60) |
| Colombia (ref.: Mexico) | -0.14  (-0.15, -0.13) | 0.36  (0.30, 0.44) | -0.14  (-0.15, -0.13) | 0.37  (0.30, 0.45) |

Table 4. Association between potential years of exposure to the CT and schooling (completed secondary or not) among participants aged 18-30 years from Mexico, South Africa, and Colombia (N= 15,431).

|  | Model 1. Schooling (completed secondary or not) | Model 2. Schooling (completed secondary or not)  Interaction with sex |
| --- | --- | --- |
|  | Marginal effect  (95% CIs) | Marginal effect  (95% CIs) |
| Years of potential exposure | 0.01  (-0.001, 0.02) | 1.02  (1.00, 1.04) |
| Sex (ref.: male) | -0.03  (-0.04, -0.01) | 0.85  (0.76, 0.97) |
| Years of potential exposure * sex (ref.: male) | N/A | 0.99  (0.97, 1.00) |
| Poverty (ref.: no poverty) | 0.22  (0.21, 0.23) | 2.23  (2.06, 2.41) |
| Birth year 1986-1988 (ref.: 1983-1985) | -0.05  (-0.09, -0.002) | 0.78  (0.55, 1.10) |
| Birth year 1989-1991 (ref.: 1983-1985) | -0.12  (-0.18, -0.06) | 0.74  (0.52, 1.04) |
| Birth year 1992-1994 (ref.: 1983-1985) | -0.16  (-0.23, -0.09) | 0.68  (0.48, 0.96) |
| Birth year 1995-1997 (ref.: 1983-1985) | -0.19  (-0.28, -0.09) | 0.82  (0.55, 1.23) |
| Birth year 1998-2000 (ref.: 1983-1985) | -0.04  (-0.16, 0.09) | 1.18  (1.37, 3.49) |
| South Africa (ref.: Mexico) | 0.45  (0.42, 0.48) | 0.37  (0.32, 0.43) |
| Colombia (ref.: Mexico) | 0.65  (0.61, 0.69) | 1.34  (1.15, 1.55) |

Model 1: Marginal effects of the association between years of potential exposure and schooling (completed secondary or not)

Model 2: the interaction between sex and years of potential exposure on schooling (completed secondary school = 1)

Table 5. Association between potential years of exposure to the CT and employment (employed in the last year or not) among participants aged 18-30 years from Mexico, South Africa, and Colombia (N= 15,431)

|  | Model 1: Employed in last year or not | Model 2. Employed in last year or not (Interaction with sex) |
| --- | --- | --- |
|  | OR  (95% CIs) | OR  (95% CIs) |
| Years of potential exposure | 0.90  (0.88, 0.91) | 0.90  (0.89, 0.92) |
| Sex (ref.: male) | 0.25  (0.24, 0.27) | 0.28  (0.24, 0.31) |
| Years of potential exposure * sex (ref.: male) | N/A | 0.99  (0.97, 1.00) |
| Poverty (ref.: no poverty) | 0.50  (0.46, 0.54) | 0.50  (0.46, 0.54) |
| Birth year 1986-1988 (ref.: 1983-1985) | 1.07  (0.79, 1.44) | 1.07  (0.80, 1.44) |
| Birth year 1989-1991 (ref.: 1983-1985) | 1.10  (0.82, 1.48) | 1.11  (0.83, 1.50) |
| Birth year 1992-1994 (ref.: 1983-1985) | 0.96  (0.71, 1.30) | 0.97  (0.72, 1.32) |
| Birth year 1995-1997 (ref.: 1983-1985) | 0.96  (0.67, 1.36) | 0.97  (0.68, 1.38) |
| Birth year 1998-2000 (ref.: 1983-1985) | 0.50  (0.33, 0.77) | 0.51  (0.34, 0.78) |
| South Africa (ref.: Mexico) | 0.34  (0.30, 0.40) | 0.34  (0.29, 0.39) |
| Colombia (ref.: Mexico) | 1.14  (0.99, 1.30) | 1.23  (0.98, 1.29) |

Model 1: The association between years of potential exposure and employment (employed in the last year or not)

Model 2: the interaction between sex and years of potential exposure on and employment (employed in the last year or not)

**Supplementary material**

**Supplementary file 1**

*Overview of CT programmes*

Colombia - Familias en Acción

Familias en Acción was launched in 2001 by the Colombian government in 700 out of 1,102 municipalities, reaching nearly 500,000 households in 2006 (Ayala, 2006). The aim was to “help reduce, overcome, and prevent poverty and income inequality, accumulate human capital, and improve living conditions of poor and vulnerable families through complementing their income”(Medellín and Prada, 2015). Cash is transferred to families with children age 0-17 years and a household income in the lowest quintile (approximately bottom 20%) of the national income distribution. Eligible families are identified from a proxy means test. When first introduced, households were transferred an average of USD 45 every month (Marulanda and Paredes, 2011). The programme is conditional on prespecified education and health requirements. The educational conditions state that children must be enrolled in either primary school (ages 7-11) or secondary school (ages 11-17) and attend 80% of school days at minimum. The health conditions state that children (ages 0-7) must attend regular check-ups at a local healthcare provider (Medellín and Prada, 2015).

South Africa – Child Support Grant

The Child Support Grant (CSG) was launched in South Africa in 1998, originally a social grant of 15 USD per month for children under 7 years of age. The CSG was designed to reduce poverty in children and focused on promoting food security but has been expanded to generally improve social investment in children’s well-being. The social grant is unconditional and targets the primary caregiver who qualifies through a means test (Lund, 2008). The eligibility criteria have changed since its inception, including an increase in the age limit from seven to eighteen years old, and alterations to the caregiver’s income threshold to take inflation into account and improve equity. In 1998, age eligibility was limited to children under 7 years old, but it was later raised to children under 9 years-old in 2003, under 11 years-old in 2004 and under 14 years-old in 2005. From January 2010, eligibility was further extended to under 18 years-old. The staggered change in age eligibility means that children born after 1 January 1994 were eligible until their 18th birthday, whereas those born before that date were not. In 2020, 12.5 million, or 63% of South Africa’s children received the CSG.

Mexico - Oportunidades

Oportunidades was established in 1997 with the dual goals of alleviating immediate suffering and breaking the intergenerational transmission of poverty by encouraging parents to invest in their children's development. Eligible families are identified from a proxy means test. Cash transfers are given to primary caregivers according to their baseline household composition for a minimum of 3 years, conditional on meeting the programme requirements. Two schemes are available: a monthly fixed stipend conditional on family members obtaining preventive medical care, intended to improve nutrition, and educational scholarships given to families of children starting the third grade in primary school, which are conditional on children attending school a minimum of 85% of the time and not repeating a grade more than twice. The grant stops when children turn 18. Beneficiary children also receive money for school supplies once or twice a year. The monthly average transfer size is about 130 USD, or 20 per cent of households’ labour income among the targeted population (ECLAC, 2016). In 2007, the budget for Oportunidades had reached $3·7 billion to cover over 5 million families.

*Supplemental Table 1.* Linear and logistic regression of effect of interaction with years of potential exposure to the CT and poverty on normalised depressive symptoms (Model 1) and binary depression (Model 2 (N= 19,878).

|  | Model 1: Normalised depressive symptoms | | Model 2: Depression (yes/no) | |
| --- | --- | --- | --- | --- |
|  | B  (95% CIs) | *p* | OR  (95% CIs) | *p* |
| Years of potential exposure | -0.002  (-0.003, -0.001) | <0.001 | 0.97  (0.95, 0.98) | <0.001 |
| Sex (ref.: male) | 0.06  (0.05, 0.06) | <0.001 | 2.09  (1.91, 2.29) | < 0.001 |
| Poverty (ref.: no poverty) | 0.02  (0.01, 0.03) | 0.004 | 1.28  (1.06, 1.53) | 0.01 |
| Years of potential exposure * poverty (ref.: no poverty) | -0.002  (-0.001, 0.001) | 0.65 | 0.99  (0.97, 1.01) | 032 |
| Birth year 1986-1990 (ref.: 1982-1985) | 0.0001  (-0.01, 0.01) | 0.99 | 1.05  (0.90, 1.23) | 0.55 |
| Birth year 1991-1995 (ref.: 1982-1985) | 0.02  (-0.01, 0.03) | 0.01 | 1.46  (1.19, 1.80) | <0.001 |
| Birth year 1986-200 (ref.: 1982-1985) | 0.02  (-0.002, 0.03) | 0.08 | 1.48  (1.13, 1.92) | 0.004 |
| South Africa (ref.: Mexico) | 0.01  (-0.002, 0.01) | 0.16 | 0.52  (0.46, 0.59) | <0.002 |
| Colombia (ref.: Mexico) | -0.13  (-0.14, -0.12) | <0.001 | 0.40  (0.35, 0.47) | <0.001 |

*Supplemental Table 2.* Linear regression for length of exposure on employment (employed in last year or not) for subgroup who were not currently in schooling (N = 10,136)

|  | Depression (yes/no) | |
| --- | --- | --- |
|  | OR  (95% CIs) | *p* |
| Years of potential exposure | 0.84  (0.79, 0.89) | <0.001 |
| Sex (ref.: male) | 0.14  (0.13, 0.15) | <0.001 |
| Poverty (ref.: no poverty) | 0.56  (0.61, 0.61) | <0.001 |
| Birth year 1986-1988 (ref.: 1983-1985) | 1.08  (0.82, 1.42) | 0.58 |
| Birth year 1989-1991 (ref.: 1983-1985) | 1.61  (1.12, 2.32) | 0.01 |
| Birth year 1992-1994 (ref.: 1983-1985) | 2.15  (1.30, 3.56) | 0.003 |
| Birth year 1995-1997 (ref.: 1983-1985) | 2.79  (1.45, 5.37) | 0.002 |
| Birth year 1998-2000 (ref.: 1983-1985) | 2.17  (0.96, 4.89) | 0.06 |
| South Africa (ref.: Mexico) | 0.39  (0.33, 0.47) | <0.001 |
| Colombia (ref.: Mexico) | 2.17  (0.96, 4.89) | 0.41 |

**References supplementary material**

ECLAC. 2016. Conditional Cash Transfer Programmes Non-contributory Social Protection Programmes in Latin America and the Caribbean [Database]. ECLAC Online observatory on Social Development. https://dds.cepal.org/bpsnc/cct

Lund F. 2008. Changing Social Policy: The Child Support Grant in South Africa. Cape Town: HSRC Press.

Marulanda B, Paredes M. 2011. Familias en Accion: A Financial Inclusion Strategy. CGAP. https://www.cgap.org/blog/familias-en-accion-financial-inclusion-strategy

Medellín N, Prada FS. 2015. How Does Más Familias en Acción Work. Best Practices in the Implementation of Conditional Cash Transfer Programs in Latin America and the Caribbean.
